# Do generalized epilepsies exhibit more attentional or executive disorders?

**DOI:** 10.1016/j.ebr.2025.100843

**Published:** 2025-12-24

**Authors:** Bertille Lacour, Vincent Heintz, Natacha Forthoffer, Serge Chassagnon, Alain Jager, Louis Maillard, Helene Brissart

**Affiliations:** a Reference Center for Cerebellar Malformations and Congenital Diseases, Department of Pediatric Neurology, Lille University Hospital, 59000 Lille, France^1^; b Reference Center for Rare Epilepsies, Department of Pediatric Neurology, Lille University Hospital, 59000 Lille, France^1^; c Hopale Foundation, Sainte Barbe Center, 62740 Fouquières-Lès-Lens, France^1^; d Department of Neurology, Nancy University Hospital, 54000 Nancy, France; e Saint-Vincent Hospital Group, Sleep and Epilepsy Center, 67000 Strasbourg, France; f Private Practice, Thionville, France; g IMoPA (UMR 7365, CNRS), University of Lorraine, Nancy, France

**Keywords:** Epilepsy, Neuropsychology, Attention, Executive funtions

## Abstract

This study aimed to characterize attentional and executive dysfunctions in patients with genetic generalized epilepsies (GGE), using specific and validated neuropsychological tools. Secondary objectives included estimating the prevalence of cognitive impairments and exploring potential clinical and psychosocial determinants. A total of 69 adult patients with GGE were enrolled in this multicentric observational study. Participants completed screening questionnaires for anxiety and depression and underwent a battery of neuropsychological tests assessing four attentional components (alertness, sustained, selective, and divided attention), executive functions (flexibility, inhibition, verbal initiation and auditory working memory). Attentional and executive impairments were highly prevalent, affecting approximately 60–64 % of the cohort. Divided attention was the most frequently impaired attentional component (44.93 %), while mental flexibility was the most commonly affected executive function (39.68 %). Selective and sustained attention were relatively preserved. Working memory manipulation was impaired in 17.4 % of patients. Cognitive performance was weakly influenced by clinical and psychosocial factors. Anxiety or depression was associated with poorer divided attention, epilepsy duration with lower selective attention, and education and number of anti-seizure medications with better performance across selective, sustained, and executive attention domains. These findings highlight the need for targeted neuropsychological assessments in GGE, prioritizing divided attention and flexibility. Since divided attention reflects both attentional control and working memory interventions focusing on working memory may offer greater clinic utility. Given the scarcity of studies on cognition rehabilitation in epilepsy, further research is needed, particularly on divided attention as a potential cognitive remediation target.

## Highlights

• Attentional and executive deficits each affect ∼ 60 % of GGE patients.
• Divided attention is the most impaired function and a marker of working memory.
• Mental flexibility is the most affected executive component (40% of patients).
• Multidimensional testing reveals distinct cognitive profiles in GGE.
• Incorporating divided attention tasks improves cognitive assessment accuracy.

## 1 Introduction

Generalized genetic epilepsy (GGE), as defined by the International League Against Epilepsy (ILAE) in 2017, constitutes a heterogeneous group including common idiopathic generalized epilepsies, developmental and epileptic encephalopathies [1]. GGE accounts for approximately 20 % of all epilepsies [2] its neuropsychological co-morbidities have been underexplored [3] and are often overlooked. This highlights the need for a deeper understanding of the cognitive impairments associated with GGE [4].

Cognitively, GGE are characterized by attentional and executive disorders [5]. Impairments in executive function (EF) are prominent [2], [6], [7], [8], with reduced psychomotor speed and executive dysfunction in GEE [9].

Attention plays a pivotal role in cognitive functioning, significantly overlapping with EF and working memory (WM) [10], [11]. Attention is often conflated with these, as its mechanisms underpin their effective functioning [12]. Furthermore, psychiatric comorbidities (e.g depression or anxiety) frequently exacerbate cognitive impairments [13], [14].

ILAE recommended to assess attention in GGE [15], but studies in this area remain scarce, particularly in adults. Methodological limitations contribute to this gap. For instance, widely used tools such as the Trail Making Test A or Coding (Wechsler Adult Intelligence Scale - WAIS IV) primarily assess processing speed, while others like the D2 Task, focus on Selective Attention in the visual modality. Many evaluations rely on non-specific tools, such as digit spans or the EPITRACK battery, which fail to comprehensively capture the different facets of attentional impairments [16], [17]. To our knowledge, only one study, which was limited to pediatric populations, has assessed all four attentional aspects (alertness, Sustained Attention, Selective Attention and Divided Attention) as defined by the Van Zomeren and Brouwer model [18], [19].

Moreover, WM is a multidimensional construct that plays a central role in many higher-order cognitive processes. It includes several components – the central executive, visuospatial sketchpad, episodic buffer, and phonological loop – that support the temporary storage, manipulation of information and more complex tasks involving dual-task coordination and executive control [20], [21], [22]. It highlights the need for specific assessment tools in order to differentiate between attentional, executive, and WM impairments.

It is also critical to consider the influence of both nonspecific and epilepsy-specific determinants on cognitive functioning, despite the inherent difficulties in differentiating one from the other [12], [16]. Factors such as mood and educational level must be accounted for, as they can influence findings. Specific determinants, including the duration and age of epilepsy onset, drug-sensitivity (1 year without seizure under treatment) and anti-seizure medication (ASM), are particularly relevant for understanding the cognitive and attentional outcomes in GGE.

The present study primarily aims to characterize executive and attentional impairments in GGE using robust and specific tools to assess attention, executive function and working memory. Secondary objectives include (1) exploring the prevalence and nature of cognitive impairments in GGE, (2) identifying potential underlying determinants. These insights are crucial for optimizing patient care.

## 2 Method

### 2.1 Participants

The multicentric prospective observational study included 69 patients from 28 years old to 59 ($\overset{¯}{x}$ = 33.5; σ = 13.6), diagnosed with GGE in the period 20/05/2020 to 06/01/2022. 39 were female (56.5 %) and 30 were male (43.5 %). 61 (88.4 %) were right-handed and 8 (11.6 %) left-handed.

### 2.2 Procedure

The cohort reflects a multicentric recruitment. 39 patients were enrolled from the Neurology Department of the University Hospital of Nancy, 21 from SELEST center in Strasbourg and 9 from Private office in Thionville. This recruitment strategy ensured the inclusion of diverse epileptic profiles, spanning both hospital-based and private-practice follow-ups. All participants have a diagnosis of GGE based on Fisher’s criteria [23].

Participants were recruited on a voluntary basis as part of their neurological follow-up. The investigating neurologists invited eligible patients to participate in the **Etude des Troubles Attentionnels chez les Patients Epileptiques (ETAPE)** study (ID-RCB n°: 2019-A02748-49), which received ethical approval from the Comité de Protection des Personnes (Protection of people committee) on 02/04/2020.

Exclusion criteria were as follows: presence of neurological diseases other than epilepsy, presence of developmental and epileptic encephalopathies based on ILAE criteria, evidence of developmental regressions temporally linked to epileptic activity and expert consensus review of clinical and EEG data, legal protection measures preventing informed consent, deprivation of liberty by judicial or administrative decision, regular use of psychoactive substances (e.g., cannabis, alcohol), major depressive syndromes (with suicidal ideation) and patients taking neuroleptics from our population.

IQ was not an inclusion / exclusion criterion based on the fact that the main objective was to assess attention and EF in a limited protocol in time. Education level was collected as a robust indicator of cognition.

After agreement to participate, participants gave their informed consent. On the assessment’s day, participants were invited to complete self-reported screening scales before undergoing a series of neuropsychological tests assessing attentional, executive function and working memory. The entire assessment lasted approximately 1 h and 15 min (appendix a).

### 2.3 Materials and interpretation

#### 2.3.1 Screening scales

Two validated self-reporting questionnaires were used to assess anxiety and depression: The **Neurological Disorders Depression Inventory for Epilepsy (NDDI-E) - French version** [24], [25] (cutoff score of 15/24). And the **Generalized Anxiety Disorder-7 (GAD-7)** [26], [27] (cutoff score of 7/21).

#### 2.3.2 Neuropsychological tests

Cognitive functions were assessed using selected subtests from validated batteries: **Test of Attentional Performance Battery Version 2.3.1 – TAP** [28], [29]. Alertness, divided attention, sustained attention, flexibility, and inhibition were administered on computer. Clinical cut-offs were based on T40 following author’s instruction. **D2-R Test** [30]: a computerized version assessed selective Attention using accuracy as the primary performance index, which balances speed and precision. The threshold is a percentile score below 5, following author’s instruction. **Literal Fluency (GREFEX Battery)** [31]: participants generated as many words as possible starting with the letter “P” within two minutes, assessing verbal initiation. The threshold is a cut-off score, following author’s instruction. **Digit Span (WAIS-IV)** [32]: Forward and backward span tests were used to evaluate working memory. The clinical cut-off was defined as a difference of 4 between forward and backward span, as this variation has been observed in a cumulative percentage of 6.8 % within the healthy population, according to the authors.

#### 2.3.3 Selection of performance indicators

For each test, specific performance indicators were chosen based on their clinical validation, authors’ suggestion, international standards and their sensitivity to deficits observed in epilepsy: alertness index, omissions (divided attention) and omission in later phases (sustained attention) to minimize errors in interpreting WM deficits, incompatibility index, flexibility performance index, accuracy index (selective attention).

#### 2.3.4 Justification of methodological choices

In neuropsychology, patients’ performance is interpreted relative to normative data from reference populations. Because deviations from expected performance depend on how each test was standardized, the cut-off values used to define impaired performance necessarily vary across instruments (according to the statistical distribution of normative scores, the size and characteristics of the normative samples, the sensitivity–specificity balance intended by authors, the type of metric provided and thresholds recommended by the original authors). Consistent with these principles, we applied the author-recommended cut-off scores for each test to ensure valid identification of impairments, as we do in clinical practice. Two types of composite scores were derived: for each cognitive function, test performances were dichotomized using validated cut-offs, and a binary composite was derived (1: impaired, 0: preserved). We additionally computed composite scores reflecting the number of impaired tests within each domain (e.g., 0–4 for attention and executive functions), providing an index of domain-specific burden. This two-level composite approach allows both the estimation of prevalence of impairment and the characterization of intra-domain cognitive vulnerability, providing a robust and clinically meaningful description of cognitive deficits in GGE [33].

### 2.4 Data analysis

Descriptive and inferential statistics were conducted using JAMOVI® to estimate the prevalence of cognitive impairments. Prevalence was determined from the binary composite indices derived from clinical cut-offs. The Shapiro-Wilk test was applied to assess the normality of the distributions in order to use Spearman’s rho correlations (p < 0.05) to examine the associations between clinical variables and cognitive outcomes. For sustained attention, a composite score was derived from omission patterns over time as described in the method so the variable was not continuous. That’s why we used Fisher’s exact test (with Cramer’s V) (p < 0.05).

## 3 Results

### 3.1 Demographic and clinical characteristics

Among our participants, educational attainment was < 12 years in 26.1 %, 12 years in 17.4 %, and > 12 years in 56.5 % (range 9–17; mean 13.49 ± 2.44). Age at epilepsy onset ranged from 1 to 56 years (mean 16.29 ± 8.85), with epilepsy duration ranging from < 1 to 62 years (mean 17.14 ± 14.78). Drug-resistant epilepsy (persistence of seizures despite administration of two appropriate ASM at effective doses that are well tolerated, either as monotherapy or combination) was identified in 20.3 %, while 79.7 % were drug-sensitive. ASM included levetiracetam, lamotrigine, valproate, brivaracetam, lacosamide, topiramate, clobazam, eslicarbazepine acetate, carbamazepine, and zonisamide; 4.35 % received no ASM, 58.0 % one ASM, 26.1 % two ASMs, and 11.6 % three ASMs. The cohort included 1 childhood absence epilepsy (CAE), 3 juvenile absence epilepsy (JAE), 15 juvenile myoclonic epilepsy (JME), 5 epilepsy with eyelid myoclonia (EEM), and 45 other GGE syndromes. Clinically significant symptomatology was observed in 18.8 % on the NDDI-E, 53.6 % on the GAD-7, and 18.8 % on both. Data were missing for up to nine participants for some subtests due to unavailable normative data for specific ages.

### 3.2 Nature and prevalence of attentional disorders

**Fig. 1:**
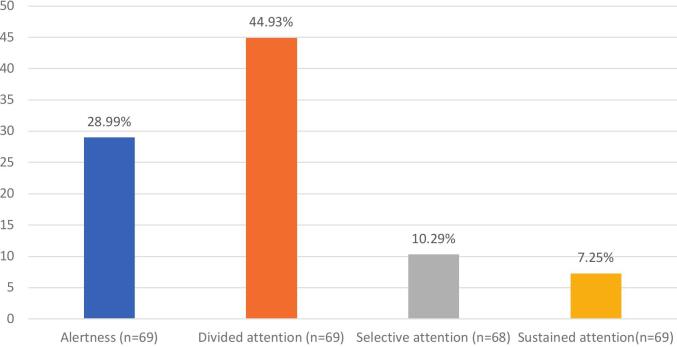
Prevalence of attentional disorders.

**Fig. 2:**
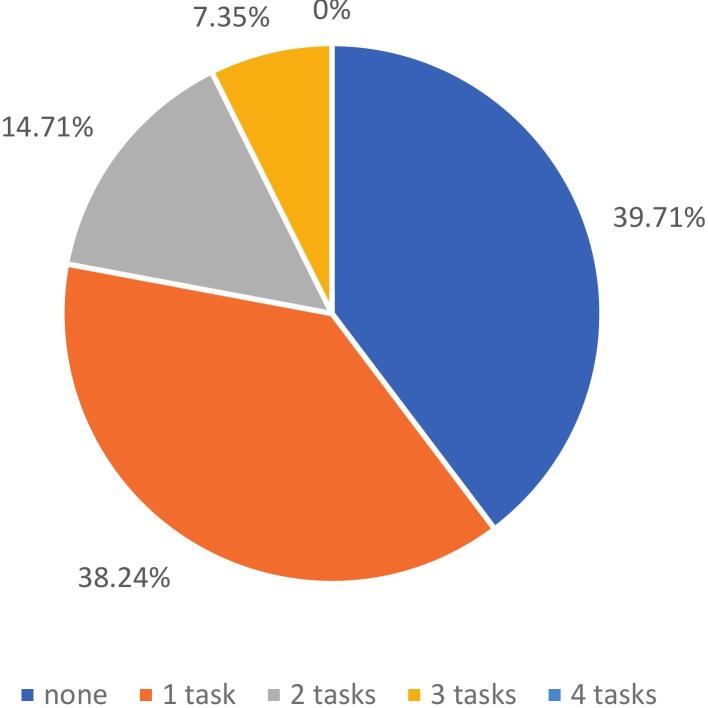
Number of failed attentional task per patient.

Divided attention was the most frequently impaired component of attention, affecting 44.93 % of participants. Alertness was impaired in 28.99 % of cases, selective attention in 10.29 % and sustained attention 7.25 % of cases (Fig. 1). It is important to note that 60.3 % of participants had at least one attentional test result indicating impairment (Fig. 2).

### 3.3 Nature and prevalence of executive disorders

**Fig. 3:**
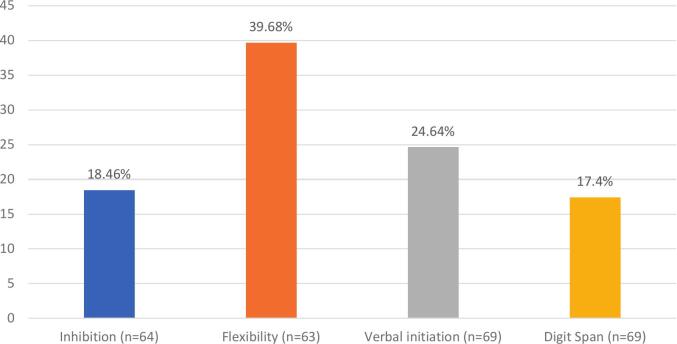
Prevalence of executive disorders.

**Fig. 4:**
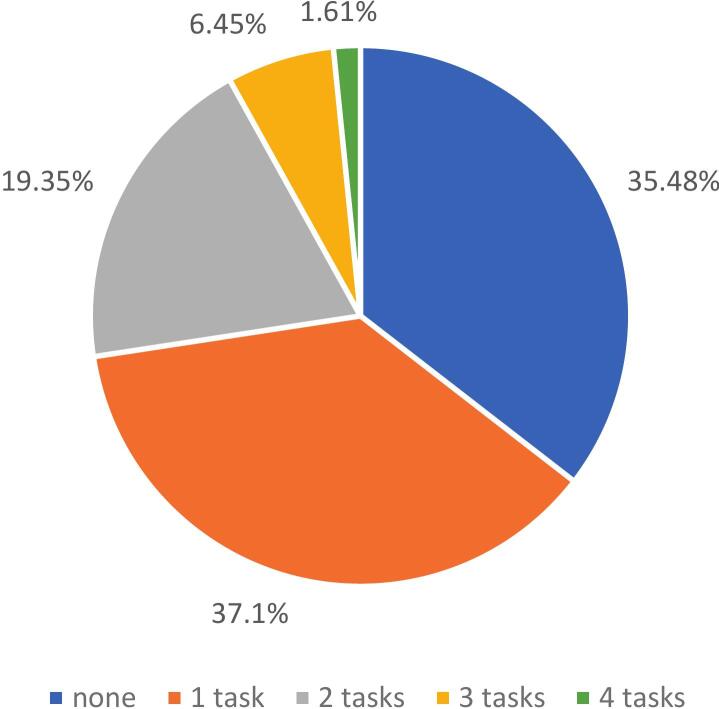
Number of failed executive task per patient.

Flexibility was the most disturbed component, observed in 39.68 % of participants. Verbal initiation was impaired in 24.64 % and inhibition in 18.46 % of cases. Finally, manipulation in auditory WM was impaired in 17.40 % of cases (Fig. 3). It is important to note that 64.52 % of participants failed at least one executive subtest (Fig. 4). Excluding working memory, 61.29 % of participants failed at least one executive subtest (appendix b).

### 3.4 Effects of factors on attentional and executive function

9 patients exhibited no cognitive impairment (appendix c, d). They displayed heterogeneous demographic and clinical profiles, sharing only right-handedness and drug-sensitivity. Higher anxiety and depressive symptomatology were both associated with poorer divided attention performance (rho = -0.256, p = 0.034; rho = -0.251; p = 0.038, respectively). Longer epilepsy duration was associated with poorer selective attention (rho = -0.298; p = 0.014). Higher educational level was associated with better performance in selective attention (rho = 0.244; p = 0.045), flexibility (rho = -0.369; p = 0.003) and verbal initiation (rho = 0.294; p = 0.014). A higher number of ASM was associated with better sustained attention performance (p = 0.019; V = 0.508). Detailed statistical results are provided in Table 1, Table2.

**Table 1:** Effects of factors on cognitive function.

Factors	Alertness n = 69 w = 0.44 p = 0.004	Divided attention n = 69 w = 0.925 p = 0<.001	Selective attention n = 68 w = 0.916 p = 0<.001	Flexibility n = 63 w = 0.923 p = 0<.001	Inhibitory n = 60 w = 0.887 p = 0<.001	Verbal initiation n = 69 w = 0.909 p = 0<.001	Digit spans n = 69 w = 0.935 p = 0.001
Duration of epilepsy Person’s r Spearman’s p (rho)	− rho = −0.230 p = 0.057	− rho = 0.032 p = 0.797	**↓** **rho =** −**0.298** **p = 0.014**	− rho = 0.058 p = 0.651	− rho = −0.173 p = 0.71	− rho = −0.72 p = 0.555	− rho = 0.074 p = 0.545
Number of ASM Person’s r Spearman’s p (rho)	− rho = −0.131 p = 0.284	− rho = 0.114 p = 0.353	− rho = 0.013 p = 0.917	− rho = −0.068 p = 0.598	− rho = −0.130 p = 0.306	− rho = −0.098 p = 0.423	− rho = 0.075 p = 0.539
Age onset epilepsy Person’s r Spearman’s p (rho)	− rho = −0.104 p = 0.397	− rho = 0.113 p = 0.357	− rho = 0.054 p = 0.662	− rho = 0.206 p = 0.106	− rho = −0.080 p = 0.531	− rho = −0.119 p = 0.332	− rho = 0.036 p = 0.769
Anxious symptomatology Person’s r Spearman’s p (rho)	− rho = 0.072 p = 0.555	**↓** **rho =** −**0.256** **p = 0.034**	− rho = −0.004 p = 0.974	− rho = −0.183 p = 0.150	− rho = −0.029 p = 0.819	− rho = −0.114 p = 0.352	− rho = 0.041 p = 0.736
Depressive symptomatology Person’s r Spearman’s p (rho)	− rho = 0.101 p = 0.413	**↓** **rho =** −**0.251** **p = 0.038**	− rho = −0.101 p = 0.413	− rho = −0.200 p = 0.116	− rho = −0.031 p = 0.810	− rho = −0.018 p = 0.883	− rho = −0.120 p = 0.325
Level of education Person’s r Spearman’s p (rho)	− rho = −0.125 p = 0.307	− rho = −0.231 p = 0.056	**↑** **rho = 0.244** **p = 0.045**	**↑** **rho =** −**0.369** **p = 0.003**	− rho = −0.010 p = 0.937	**↑** **rho = 0.294** **p = 0.014**	− rho = −0.146 p = 0.231

**Table2:** Effects of factors on sustained attention.

Factors	Sustained attention n = 69 (Fisher’s exact Test and Cramér’s Value)
Duration of epilepsy (<15 VS 15y and more)	− p = 0.389 V = 0.125
Number of ASM	**↑** **p = 0.019** **V = 0.508**
Age onset epilepsy <15 yo, 15 yo, > 15yo U Mann-Whitney bilateral except for sustained attention	− p = 1.000 V = 0.044
Anxious symptomatology	− p = 0.363 V = 0.148
Depressive symptomatology	− p = 0.235 V = 0.151
Drug resistant epilepsy	− p = 0.575 V = 0.141
Level of education (<12y, 12 y, > 12y)	− p = 0.824 V = 0.149

## 4 Discussion

GGE patients exhibit a similar prevalence of attentional and executive disorder, with 60–64 % of patients affected by each, underscoring the critical need for targeted cognitive assessments and interventions.

Divided attention is the most impaired cognitive function (44.93 %), followed by alertness (28.99 %), selective attention (10.29 %) and sustained attention (7.25 %). In EF, flexibility (39.7 %), initiation (24.6 %) and inhibition (18.5 %) were most affected. Manipulation in auditory WM was impaired in 17.40 % of participants. While WM impairments were less frequent, the prominent issue lies in divided attention, which can be considered a higher-level component of working memory, particularly when engaging in dual-task performance. According to Baddeley’s model of WM [20], [21], [22], the central executive plays a key role in managing dual-task, such as dividing attention. Traditional measures, such as backward span tasks, might not fully capture the complexities of working memory. We recommend that clinical assessments of WM should include dual-tasks, providing a more comprehensive assessment of cognitive function. The impact of impaired divided attention is particularly important as it can have significant consequences for patients such as increased accident risk, decreased work efficiency, strained social interactions, and reduced caregiving capacity.

Alertness was impaired in 28.99 % of GGE patients, reflecting a delay in their ability to engage and react promptly. Selective attention and sustained attention were generally preserved (impairments in only 10.29 % and 7.25 % of cases, respectively) suggesting that single-task focus is relatively intact.

Flexibility emerged as the most impaired EF (39.68 %), followed by initiation (24.64 %) which is consistent with prior findings [5]. Inhibition was less affected (18.5 %).

GGE patients exhibited comparable impairments in attentional and EF, supporting a strong interconnexion between these systems. EF, attention and WM rely on attentional process [12]. The use of a multidimensional assessment [15] revealed distinct cognitive profiles, underlying the sensitivity. These results support a more integrated executive-attentional model, including WM, and underline the need to assess each subcomponent individually.

Using the IC-Code criterion (failure of at least 2 tests per domain) [34] fewer patients met the threshold for a domain deficit (22.06 % attention, 27.41 % executive). Relevant subtests were divided attention and alertness for attention and flexibility and initiation for EF. It should be consider required for each GGE patients’ assessment while others can be considered complementary. WM, overlapping attention and EF should be firstly assessed by divided attention. Notably, even a single failure may indicate difficulties, highlighting the importance of identifying subtle cognitive deficits.

Mood is known to influence cognition [35], [36]. In our study, anxious or depressive symptomatology showed a modest effect on divided attention, unlike previous studies [37], likely due to group imbalances and the use of screening questionnaires that may miss subclinical symptoms. ASM can affect cognition [38] negatively by side the addition of molecules [39] or positively by reducing seizures [40], [41]; in this study, we only had a modest size effect in sustained attention. Age of onset and epilepsy duration were unlinked to cognitive performance, except for a negative effect on selective attention aligning with previous studies [42]. Higher education was associated with better performance in some functions but the effect sizes remained moderate. Globally, group imbalances limited conclusions, suggesting future studies should recruit predefined subsamples based on modulators (e.g., patients treated with none, one, two, three or more ASM, …). Overall, while these findings highlight relevant trends, the moderate effect sizes suggest that clinical impact is limited and that additional factors likely contribute to cognitive variability in GGE patients.

Nine participants showed no cognitive impairments, sharing only right-handedness and drug-sensitivity, suggesting possible genetic influences, as reported in juvenile myoclonic epilepsy [42] and idiopathic generalized epilepsies [5].

A few limitations arise. First, the identified factors showed variable and isolated effect, suggesting additional unmeasured influence; the limited sample size precluded use of a weighted generalized linear model to estimate predictor contributions, underlying the need for larger cohorts. Second, given the exploratory design and sample size, multiple comparisons were performed without formal correction, calling for cautious interpretation of the results. Third, reliance on normative data rather than a control group enhances generalizability but limits direct comparisons to specific populations, while aligning with typical clinical practice. Then, inclusion of EEM reflects the 2022 ILAE reclassification, although the study began in 2020. Finally, fatigue and sleep were not measured, though both affect cognitive performance [43].

## 5 Conclusion

This study demonstrates that attentional and executive dysfunctions are highly prevalent among GGE patients, each affecting approximately 60 % of the cohort. Divided attention was identified as the most impaired attentional component, while mental flexibility was the most impaired executive function.

We recommend including divided attention assessments in standard neuropsychological evaluations, as they provide a more accurate indicator of WM. Because WM shows greater responsiveness to cognitive remediation than attention alone, addressing deficits in divided attention may improve functional outcomes and quality of life for patients with GGE. Given the limited research on cognitive rehabilitation in epilepsy, further studies are needed to investigate WM-focused interventions, particularly targeting divided attention, in this population.

## Ethical statement

This study was approved by the Comité de Protection des Personnes (CPP; Protection of People Committee) on April 2, 2020 (ID-RCB n° 2019-A02748-49). Participants were recruited voluntarily as part of their neurological follow-up, and all provided informed consent prior to inclusion. The study was conducted in accordance with the Declaration of Helsinki.

## CRediT authorship contribution statement

**Bertille Lacour:** Writing – original draft, Investigation, Data curation. **Vincent Heintz:** Investigation, Formal analysis, Data curation. **Natacha Forthoffer:** Writing – review & editing, Validation, Resources, Investigation. **Serge Chassagnon:** Resources, Investigation. **Alain Jager:** Resources, Investigation. **Louis Maillard:** Writing – review & editing, Validation, Resources, Investigation. **Helene Brissart:** Writing – review & editing, Validation, Supervision, Resources, Project administration, Methodology, Investigation, Conceptualization.

## Declaration of competing interest

The authors declare that they have no known competing financial interests or personal relationships that could have appeared to influence the work reported in this paper.
