# Supplementary material for: Do generalized epilepsies exhibit more attentional or executive disorders?
Source: Epilepsy Behav Rep. 2025 Dec 24;33:100843. doi: 10.1016/j.ebr.2025.100843 (PMC12950469; doi:10.1016/j.ebr.2025.100843)
Supplement: Supplementary Data 1 [file mmc1.docx]

Appendices:

1.
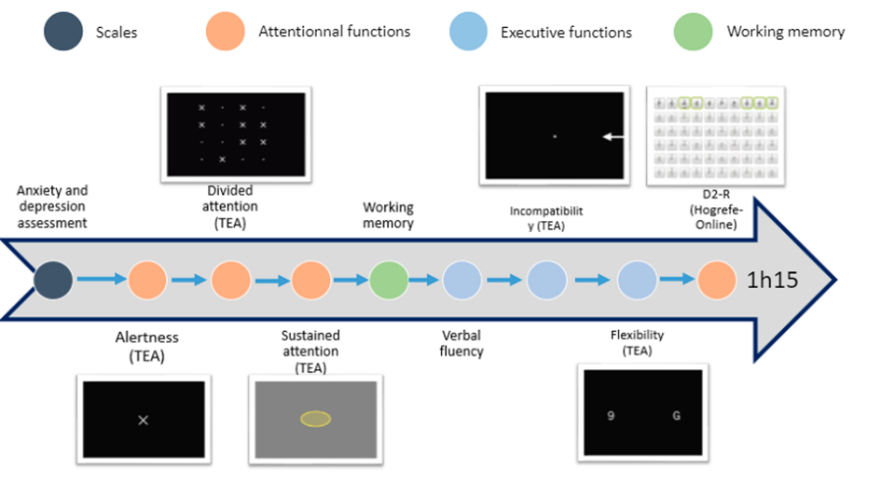
Schema reflecting the study design
2. Number of failed executive tasks per patient, excluding working memory
3.
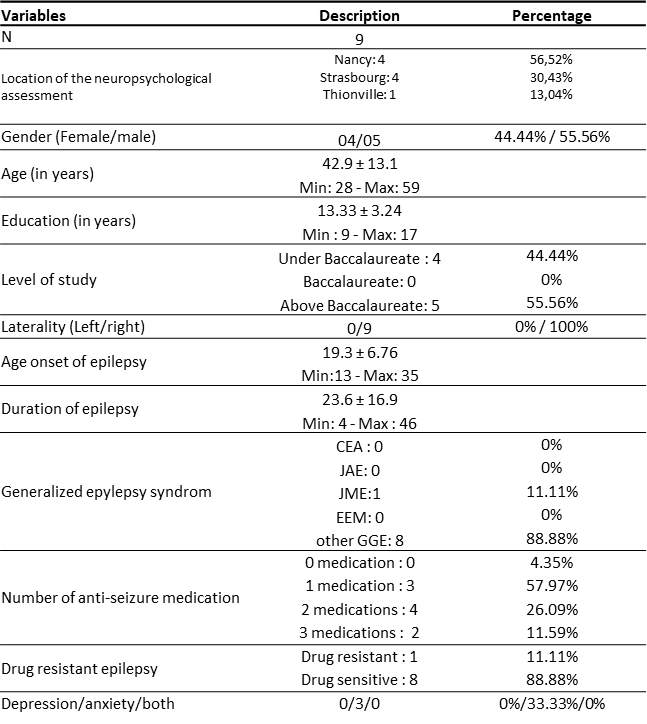
Demographic data of non-disturbed participants

1.
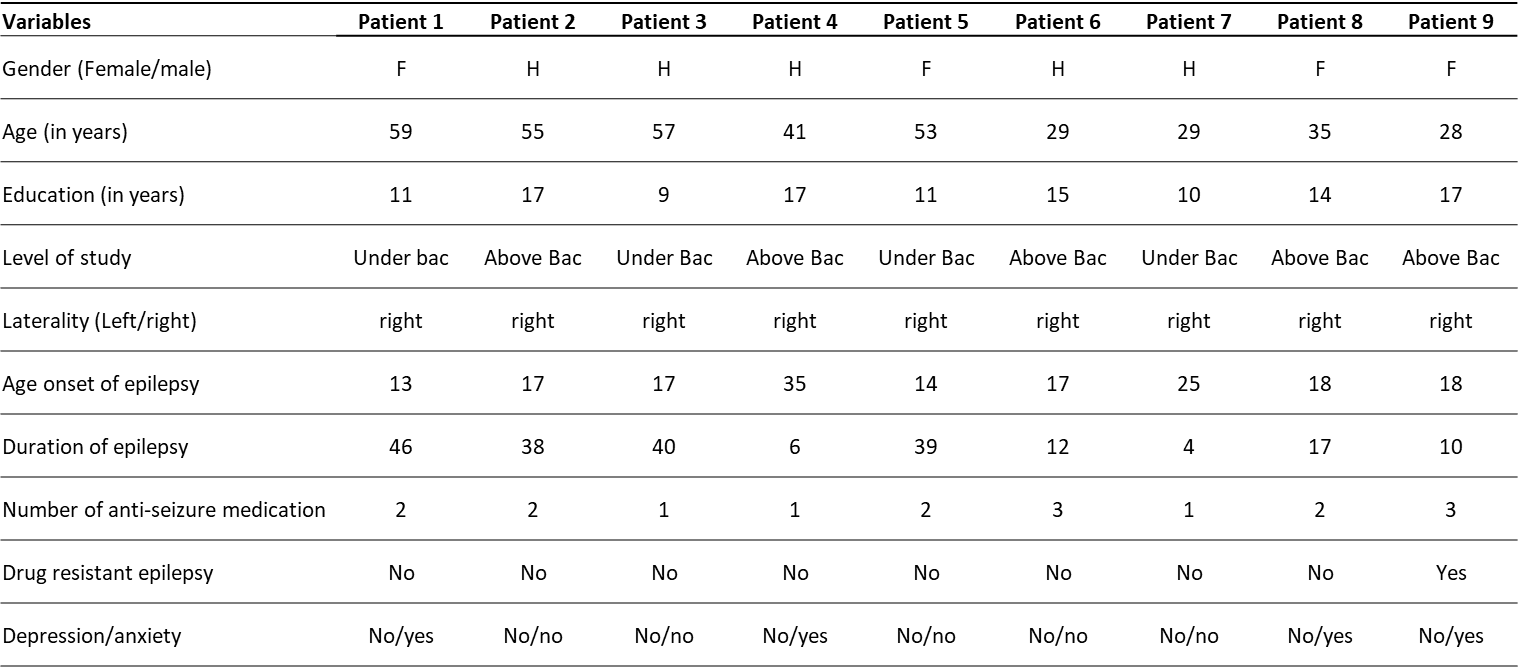
Detailed profile of non-disturbed participants
